# Regarding: “exploring the key ingredients and mechanisms of Banxia Xiexin decoction for the treatment of polycystic ovary syndrome based on network pharmacology and experimental validation”

**DOI:** 10.1080/07853890.2025.2609417

**Published:** 2025-12-29

**Authors:** Junya Shen

**Affiliations:** Affiliated Hangzhou First People’s Hospital, Westlake University School of Medicine, Hangzhou, China

We read with interest the study by Hai et al. on the mechanisms of Banxia Xiexin decoction (BXD) in treating polycystic ovary syndrome (PCOS) [1]. The authors provide a valuable contribution by applying network pharmacology *in silico* predictions and validating them with *in vivo* experiments, offering important insights into this traditional formula.

To build upon this valuable work, we believe two key methodological aspects warrant further discussion to enhance the interpretation of these findings. First, the study design compares the effects of the complete BXD formula against three putative active ingredients. This approach, while informative, raises concerns regarding pharmacokinetic comparability. The *in vivo* exposure (ADME) of these compounds when administered within the complex matrix of the entire decoction is unlikely to be equivalent to their exposure when administered in isolation. Therefore, interpreting the therapeutic differences between these groups becomes challenging, and the foundational, synergistic principles of holistic traditional formulas may be overlooked [2,3].

Second, the study insightfully identifies an association between BXD’s effects and the PI3K-AKT/MAPK pathways. These findings are important, but they currently establish a correlation rather than a definitive causal link. To substantially strengthen this mechanistic claim, a crucial next step would be functional validation, such as a blockade assay. Demonstrating that BXD’s therapeutic effects are attenuated or abolished by specific pathway inhibitors would provide the necessary evidence to confirm that these pathways are *dependent* drivers of the observed efficacy [4,5].

In summary, Hai et al. have provided an important foundation for understanding BXD. We suggest that future studies would be greatly enhanced by addressing the pharmacokinetic complexities of the holistic formula versus its components and by incorporating functional assays to confirm causal pathways. These steps would help solidify the interpretation of this work and guide the future pharmacological development of traditional medicines.

## Data Availability

Data sharing is not applicable to this article as no data were created or analysed in this study.
